# Clinical Characteristics of Diabetic Ketoacidosis in Children with Newly Diagnosed Type 1 Diabetes in Addis Ababa, Ethiopia: A Cross-Sectional Study

**DOI:** 10.1371/journal.pone.0169666

**Published:** 2017-01-30

**Authors:** Helen Siyoum Atkilt, Muluken Gizaw Turago, Balewgizie Sileshi Tegegne

**Affiliations:** 1 Bole Health Center, Addis Ababa, Ethiopia; 2 Department of Preventive Medicine, School of Public Health, College of Health Sciences Addis Ababa University, Addis Ababa, Ethiopia; 3 Department of Public Health, College of Health and Medical Sciences, Haramaya University, Harar, Ethiopia; Universita degli Studi di Perugia, ITALY

## Abstract

**Background:**

Diabetic ketoacidosis (DKA) is one of the most serious acute complications of type 1 diabetes (T1D) and the leading cause of morbidity and mortality in children with T1D. This study was aimed at assessing the prevalence and associated factors of DKA in children with newly diagnosed T1D in Addis Ababa.

**Methods:**

A hospital based cross-sectional study was conducted in selected hospitals in Addis Ababa. Children below the age of 12 years with DKA who were admitted to the pediatric ward in the selected hospitals between January 2009 and December 2014 and the residence of Addis Ababa were included. DKA was defined as children below the age of 12 years who have blood glucose level ≥250mg/dl, ketonuria, and ketonemia and diagnosed being T1D patient for the first time. Descriptive statistics was performed using frequency distribution, mean, median, tables, and graphs. Logistic regression analysis was used to identify independent factors associated with the prevalence of DKA in children with newly diagnosed T1D.

**Results:**

Of 395 DKA patients who were hospitalized during the five-year period, 142(35.8%) presented with DKA at first diagnosis of diabetes. On the other hand 253 (64.2%) children with DKA had longstanding T1D. Independent factors associated with DKA include: Age category 2–4.49years, 7–9.49 years and ≥9.5years (Adjusted odd ratio (AOR) = 3.14[1.21,8.06]), 3.44(1.39,8.49) and 4.02(1.68,9.60), respectively); parents’ knowledge on the sign and symptoms of DKA (AOR = 0.51[0.27, 0.95]); sign and symptoms of DKA before the onset of DKA (AOR = 0.35[0.21, 0.59]) and infection prior to DKA onset (AOR = 3.45[1.97, 6.04]).

**Conclusions:**

The overall proportion of children diagnosed with DKA and new onset of T1D in Addis Ababa was high. In particular, children between 9–12 years of age and children whose parents did not know the sign and symptoms of DKA had a high risk of DKA at primary diagnosis of T1D.

## Introduction

Diabetic ketoacidosis (DKA) is one of the most serious acute complications of type1 diabetes mellitus (T1D)P[1]. The International Society for Pediatric and Adolescent Diabetes define DKA as a blood glucose level >11mmol/l (≈200 mg/dl), Venous PH <7.3 or bicarbonate< 15mmol/L, ketonemia, and ketonuria[2]. DKA is a medical emergency that requires treatment and monitoring for multiple metabolic abnormalities and vigilance for complications.It is the common cause of death and permanent disability in children and adolescents with new onset diabetes[1,3].

Every year, worldwide, approximately 79,100 children under 15 years of age develop T1D. Up to 80% of these young people already have DKA when they are diagnosed with diabetes. There is a wide geographic variation in the frequency of DKA at the onset of diabetes [3,4]. The mortality rate for DKA ranges from 2 to 5% in developed countries and 6 to 24% in developing countries[4]. The mortality rate of DKA in developing countries is higher due to the higher rates of infection, protein energy malnutrition, less developed medical services and delay in health seeking behavior[5,6].

Geographical factors like climate may influence the number of people affected with DKA. Countries nearer to the equator have a high prevalence of DKA due to hot climates which lead to more rapid dehydration and onset of hyperglycemia particularly in young children [7].

Many children in sub-Saharan Africa are diagnosed with diabetes only when they present with DKA, which quite often is misdiagnosed. In many sub-Saharan African countries, diabetic patients just die because health care facilities are overstretched by even more urgent healthcare needs, HIV/AIDS, malaria and other tropical diseases[8–10].

In developing countries,where medical services are less developed, the risk of dying from DKA is greater and children may die before they receive treatment[11].The actual incidence of DKA in Sub-Saharan Africa is unknown, but unlike the western country DKA is uniquely frequent among type 2 diabetes ([8]). Some observational studies in Africa suggested a high prevalence of DKA at diagnosis, which ranges 77.1% to 88% [10,12].

In Ethiopia like other African countries, the prevalence and risk factors of DKA in children with newly diagnosed T1D is not well studied. Without the knowledge of the magnitude and associated risk factors of DKA in children the preventable associated risk factors will not be handled and this leads to increase the magnitude of DKA in children. Hence, the aim of this study was to assess the prevalence and associated risk factors of DKA in children with newly diagnosed T1D in Addis Ababa, Ethiopia.

## Materials and Methods

### Study design

A hospital-based cross-sectional study was conducted using routine hospital data and interview in three different hospitals (Black Lion, Yekatit and Zewditu hospital) in Addis Ababa.

### Study setting and participants

This study was conducted in Addis Ababa from January 1 to March 30, 2015. Addis Ababa is the capital and largest city of Ethiopia. Based on the 2007 Census projection conducted by the Central Statistical Agency of Ethiopia (CSA), in 2015 Addis Ababa has a total population of 3,273,001, of whom 1,551,000 are male, and 1,722,001 female; all of the population is urban inhabitants [13]. Children below the age of 12 years with DKA who were admitted to pediatric wards in the selected hospitals between January 2009 and December 2014 and the residence of Addis Ababa were included. Black Lion, Yekatit and Zewditu hospital were selected purposely because they had higher pediatric patient flow. During the five years study period, a total of 715 children were admittedto the selected hospitals with DKA (300 in Black Lion, 210 in Yekatit and 205 in Zewditu). To calculate the adequate minimum sample size we took the magnitude of DKA from a study conducted in Tikur Anbesa specialized hospital which was 80% [11]. We have considered 95% confidence level, 4% margin of error (d) and 10% non-response rate. Based on the calculated sample, we included 421 children for analysis in this study.

### Data collection procedures

The dependent variable for this study was DKA in newly diagnosed children, and it was defined as children between 0–12 years who have blood glucose level ≥ 250mg/dl, ketonuria and ketonemia and diagnosed being T1D patient for the first time. The independent variables were socio-demographic characteristics and clinical factors like family history of DM, knowledge of the sign and symptoms of DM/DKA and others.

A checklist that measures the socio-demographic characteristics and clinical information of DKA was used to collect the data. Data which are not included in the patient card were obtained from the parents/guardians when they came with the child in the selected hospital for follow up. The patients were followed up with the frequency of visit of 1–3 months. Data was collected using systematic random sampling with ak value of two which was calculated by dividing the number of DKA patient in each hospital divided by the number of sample size allocated to each hospital. The first chart was selected randomly.

### Statistical analysis

Data was entered into EpiInfo version7 statistical software and exported to SPSS (statistical package for the social sciences) version 21. The data were examined for outliers and checked accordingly. Descriptive analysis was performed using frequency distribution, mean, median, tables and graph. A bivariate analysis was carried out using crude odds ratio (COR) to assess the relationship between dependent and independent variables. The significance of the relationship between dependent and independent variables was checked using p-value and p< 0.05 was determined as a significance relationship. Moreover, multivariate analysis was used to identify the associated risk factors by controlling possible confounding factors using logistic regression. All variables in the bivariate analysis were included in the multivariate analysis and variables were entered hierarchically to fit the logistic regression model.

### Ethical considerations

Ethical clearance for this study was obtained from the Review Ethics Committee of School of Public Health at Addis Ababa University. As the study was conducted through review of medical records and interview, the study participants were informed about the purpose of the study and the importance of their participation in the study. And also they were informed as they could not participate; stop at any time in between; skip or decline to answer questions if they felt uncomfortable without losing the benefit thatwould get in the institution. Their participation was purely on voluntary. Moreover, no personal identifiers were used on the data collection form. The recorded data were not accessed by a third person and were kept confidentially. The mother/caregiver of the children had signed the informed consent sheet.

## Results

A total of 421 charts from children below the age of 12 years who were diagnosed with DKA between January 2009 and December 2014 were reviewed. Of 421 charts, we found 395 under 12 children who came for the refill (follow-up) and answered the socioeconomic and clinical risk factors that are not included in the chart, making the response rate of 93.8%. Among a total of 395 patients with DKA, who were hospitalized during the five year period under review, 142(35.8%, 95% CI [31.6%, 40.8%]) were newly diagnosed. Of 142 newly diagnosed DM children at the primary diagnosis of DKA, more than half, 77(55.9%) were males. The mean age at presentation was 7.08±3.8 years (7.4 ± 3.74 years for boys and 6.66 ± 3.85 years for girls). Of 395 children the marital status of the mother/father/guardian with whom the child live; 330(83.5%) were married, 36(9.1%) were separated, 15(3.8%) were widowed, and the rest were single. The mean and standard deviation of family income were 2569±1216. The majority, 182(46.1%) of children were from Orthodox Christian religion family followed by, 113 (28.5%) Muslims and 60(15.2%) Protestants. Of the total respondents; majority completed at least high school grades and above (100(25.3%) were above grade 12, 93(23.5%) were 9–12). Of 395 children’s mother/caregiver; more than half were a housewife, 137(34.7%) and civil servant, 92(23.3%) by occupation (Table 1).

**Table 1:** Socio-demographic characteristics of the subjects.

Variables	Category	Frequency N = 395	Percent
Sex	Male	221	55.9
	Female	174	44.1
Age category	<2	39	9.9
	2.0–4.49	75	19
	4.5–6.99	46	11.6
	7.0–9.49	105	26.6
	> = 9.5	130	32.9
Religion	Orthodox	182	46.1
	Muslim	113	28.5
	Protestant	60	15.2
	Catholic	20	5.1
	Others	20	5.1
Ethnicity	Amhara	125	31.6
	Tigre	62	15.7
	Oromo	126	31.9
	Gurage	50	12.7
	Others	32	8.1
Mother/caregiver education	Unable to read & write	35	8.9
	Read and Write	74	18.8
	Grade 1–8	93	23.5
	Grade 9–12	93	23.5
	Above 12	100	25.3
Marital status of the mother/caregiver	Single	14	3.6
	Married	330	83.5
	Separated	36	9.1
	Widowed	15	3.8
Mother/caregiver occupation	Unemployed	28	7.1
	Civil servant	92	23.3
	Student	11	2.8
	Housewife	137	34.7
	Daily labor	57	14.4
	Merchant	58	14.7

From this study one-third of the children 131(33.2%) had first-degree relatives with diabetes. Less than half (43.0%) of the parents of children knew about the sign and symptoms of DM/DKA. The result of this study also showed the majority of mothers/caregiver’s had knowledge on polydipsia and polyuria as sign and symptoms of DM/DKA (41.1% and 36.2% respectively). In this study the frequently reported presenting symptoms of DKA were vomiting (31.9%) abdominal pain (19.8%) dry mucous membrane (16.3%) and polydipsia (11.9%).

The study showed 108(27.3%) children had an infection before the onset of DKA. Of these, 8.2% of children had dysuria, fever 7.7% and 1.4% of children had skin lesion before DKA (Table 2).

**Table 2:** Clinical characteristics of the subjects.

Variable	Category	Frequency N = 395	Percent
Child first-degree relatives with DM	Yes	131	33.2
	No	264	66.8
Parent’s knowledge about the sign and symptoms of DM/DKA	Yes	170	43
	No	225	57
Sign and symptoms of DKA before the onset of DKA	Yes	253	64.1
	No	142	35.9
Which sign and symptoms of DKA	Polyuria	12	4.8
	Polydipsia	38	15
	Weight loss	36	14.2
	Polyuria & Polydipsia	117	46.2
	Polyuria & Weight loss	38	15
	Polydipsia & weight loss	12	4.8
Infection before the onset of DKA	Yes	108	27.3
	No	287	72.7

Bivariate logistic regression analysis was done to identify the relation between DKA and the associated risk factors. In this analysis age of the child, family history of DM, preceding sign and symptoms DKA/DM, mothers’ knowledge on sign and symptoms of DM/DKA and preceding infection found to have a significant association with DKA at primary diagnosis of T1D.

In multivariate analysis, the independent variables that were not significant in the bivariate analysis like sex, marital status, and family income were not significant in the multivariate analysis too. Accordingly, knowledge of the sign and symptoms of DM/DKA, preceding sign and symptoms of DM/DKA, prior history of infection and age found to be statistically significant factors for developing DKA at the first onset of T1D. Compared to under-2- year old children; the age group 2–4.49 years and 5–9.49 years had three times and 2.6 times higher odds of developing DKA in newly diagnosed children (AOR = 3.13[1.22, 8.06]), 2.61[1.40, 8.50] respectively). Similarly, older children (≥9.5 years) also had four times higher chance of developing (AOR = 4.02[1.69, 9.60]) as compared to children less than two years (Table 3).

**Table 3:** Factors associated with diabetic ketoacidosis.

Variables		DKA		COR (95%CI	AOR (95%CI)
		Yes	No		
Age category	< = 2 year	22	17	Ref.	Ref.
	2–4.49	24	51	2.75(1.27,6.10)	3.13(1.21,8.06)*
	4.5–6.99	20	26	1.68(0.71,3.98)	2.61(0.93,7.29)
	7–9.49	41	64	2.02(0.25,0.96)	3.44(1.39,8.49)*
	>9.5	35	95	3.51(1.67,7.38)	4.02(1.68,9.60)*
Mother/caregiver education status	Unable to read & write	20	15	Ref.	Ref.
	Read and Write	30	44	1.96(0.86,4.42)	1.71(0.62,4.70)
	Grade 1–8	35	58	2.21(1.0,4.87)	1.61(0.53,4.90)
	Grade 9–12	32	61	2.54(1.2,5.60) 4.0(1.78,8.97)	1.80(0.58,5.50)
	Above 12	25	75		3.03(0.92,10.0)
Family income	< = 1000 birr	6	13	Ref.	Ref.
	10001–2200	67	90	0.62(0.22,1.72)	0.36(0.10,1.30)
	2201–3400	33	85	1.19(0.42,3.34)	0.69(0.18,2.60)
	3401–3600	30	50	0.77(0.26,2.24)	0.31(0.07,1.23)
	>3600	60	15	1.15(0.29,4.46)	0.4(0.07,2.20)
Having first degree relatives with DM	Yes	38	93	0.63(0.40,0.90)	0.77(0.40,1.52)
	No	104	160	Ref.	Ref.
	Parent’s knowledge about the sign &	Yes	46	124	0.49(0.32,0.76)	0.51(0.27,0.95)*
Symptom	No	96	129	Ref	Ref.
of DM/DKA					
Symptoms and sign of DKA before the onset of DKA	Yes	181	72	Ref.	Ref.
	No	72	70	0.41(0.27,0.63)	0.35(0.21,0.59)*

N.B. Ref. is designated for reference

*statistically significant at α = 0.05.

The final model included the following variables: Age category, mothers’ or caregivers’ educational status, family income, having first degree relative with DM, parents’ knowledge of sign and symptom of DM/DKA, infection before the onset of DKA and sign and symptoms of DKA before onset of DKA.

Children who their parents knew the sign and symptoms of DM/DKA had 49% times less likely chance to develop DKA at primary diagnosis of DM than children who their parents did not have knowledge about the sign and symptoms of DM controlling for the rest of the independent variables. Children who had first-degree relatives with DM had a significant association with new-onset DM/DKA in bivariate analysis, but it was not significantly associated in multivariate analysis adjusting for the other independent variables. The odds of developing new onset DM with DKA was 3.45 (AOR = 3.455[1.97–6.04]) times higher in children who had an infection before 1–2 week of DKA than who did not have the sign and symptoms of infection adjusting for the rest of independent variables (Table 3).

## Discussion

This study revealed over one-third of children in the present study manifested with DKA at first diagnosis of diabetes. It is not surprising as a similar prevalence has been reported in a previous study in Italy [14] and Iran (24%) [15]. Another systematic review also reported consistent findings in Finland (22%) and Hungary (23%) [7]. However, some studies have reported higher prevalence, UAE(80%), Romania(67%),Taiwan(65%)[7], South Africa[16] all reflecting the well-known wide geographic variation in frequency of DKA at the onset of pediatric diabetes mellitus. Differences in study population might explain the differing in magnitude, the background prevalence of diabetes in the given population, presence or absence of family history ofT1D, socioeconomic status, delayed diagnosis and treatment as well as the definition of DKA used in the particular study[17].

Another study conducted in black lion hospital, Ethiopia [11] found the prevalence of DKA to be 80%, which was higher than our study. The magnitude might be overestimated due to severe cases are referred to black lion hospital. Another retrospective cross-sectional study carried out in Benin teaching hospital found the frequency of DKA in newly diagnosed children to be 77.1%[10], and this percentage is higher compared to our study which might be this study included under 15 years children for extended study periods.

A study conducted in Germany did not find a significant association between parental education and development of DKA [18], similar to this study our research did not find a significant association.

In a systematic review, three studies examined the effect of family income, and two European studies found that household income had no a significant effect on risk of presenting in DKA, and this result is consistent with our results [19]. This might be due to the reason that even though parents who get higher income and having a higher level of education they might not be aware of DM/DKA and seek medical advice earlier.

Marital status of the child mother had no significant association in developing DKA in newly diagnosed T1D children both in bivariate and multivariate analysis. Marital status of the child parent’s is an indirect predictor of DKA. In single, divorced and widowed mother the family income is supposed to be lesser than in married, but family income had no significant association with DKA so do the child parent’s marital status.

In agreement with our study a German study which adjusted for age, sex, having a single parent, and social status also failed to show a significant association with a family history of either type1or type2 diabetes in siblings, parents, or grandparent [19]. Although having a first degree relative with T1D decreased the frequency of DKA in three studies; it did not predict a diagnosis of new onset diabetes before progression toDKA. This might be because it is very difficult to find the classical symptoms of DKA in young children which are polyuria, polydipsia, and weight loss so the parents of the child might not consider the other sign and symptoms of DKA as the sign of DKA.

In this cross-sectional study, the odds of developing DKA in newly diagnosed T1D children was 49% lower for children whose parents’ knew the sign and symptoms of DM/DKA than parents’ who didn’t know the sign and symptoms of DM. This is because parents’ who know the sign and symptoms of DM might seek health care before their children develop DKA.

Three studies in the systematic review by Usher Smith JA et al.[19]included data on the effect of a previous infection or febrile illness. In two of the included studies, a history of infection or febrile illness was associated with an increased risk of DKA. Similar to this our study found the odds of children who had infection 1–2 weeks before DKA at the first onset of DM was 3.45 times higher than children who did not have an infection before DKA at thefirst onset of DM.Infection is known to cause inflammation, pro-inflammatory cytokine release, and a counter-regulatory response that collectively lead to insulin resistance and metabolic decompensation[19].

This study has significant limitations. First, we could not be able to calculate the prevalence of DKA among children with T1D due to lack of data on the total number of T1D. Second, the definition used for DKA in this study was not consistent with ISPAD. Hence, due to methodological differences the comparison with other studies was challenge full. Third, because of cross-sectional nature of the study, it was not possible to determine the temporal relationship of independent variables on the development of DKA. Finally, the inclusion of only the three government hospitals might hinder the actual estimation of DKA.

## Conclusions

The overall proportion of children diagnosed with DKA and new onset ofT1Dwas high. In particular, children between 9–12 years of age had a high risk of DKA at onset. Children’s age, knowledge of sign and symptoms of DKA, sign, and symptoms of DKA before the onset of DKA and infection before DKA were found to be the significant explanatory variable of DKA in children with newly diagnosed T1D.
